# Clustering of known low and moderate risk alleles rather than a novel recessive high‐risk gene in non‐BRCA1/2 sib trios affected with breast cancer

**DOI:** 10.1002/ijc.33039

**Published:** 2020-05-30

**Authors:** Florentine S. Hilbers, Peter J. van ‘t Hof, Caro M. Meijers, Hailiang Mei, Kyriaki Michailidou, Joe Dennis, Frans B. L. Hogervorst, Petra M. Nederlof, Christi J. van Asperen, Peter Devilee

**Affiliations:** ^1^ Department of Human Genetics Leiden University Medical Centre Leiden The Netherlands; ^2^ Sequence Analysis Support Core Leiden University Medical Centre Leiden The Netherlands; ^3^ Centre for Cancer Genetic Epidemiology, Department of Public Health and Primary Care University of Cambridge Cambridge UK; ^4^ Biostatistics Unit The Cyprus Institute of Neurology and Genetics Nicosia Cyprus; ^5^ Department of Pathology The Netherlands Cancer Institute Amsterdam The Netherlands; ^6^ Department of Clinical Genetics Leiden University Medical Centre Leiden The Netherlands; ^7^ Department of Pathology Leiden University Medical Centre Leiden The Netherlands

**Keywords:** breast cancer, exome, polygenic, recessive, susceptibility

## Abstract

Breast cancer risk is approximately twice as high in first‐degree relatives of female breast cancer cases than in women in the general population. Less than half of this risk can be attributed to the currently known genetic risk factors. Recessive risk alleles represent a relatively underexplored explanation for the remainder of familial risk. To address this, we selected 19 non‐*BRCA1/2* breast cancer families in which at least three siblings were affected, while no first‐degree relatives of the previous or following generation had breast cancer. Germline DNA from one of the siblings was subjected to exome sequencing, while all affected siblings were genotyped using SNP arrays to assess haplotype sharing and to calculate a polygenic risk score (PRS) based on 160 low‐risk variants. We found no convincing candidate recessive alleles among exome sequencing variants in genomic regions for which all three siblings shared two haplotypes. However, we found two families in which all affected siblings carried the CHEK2*1100delC. In addition, the average normalized PRS of the “recessive” family probands (0.81) was significantly higher than that in both general population cases (0.35, *P* = .026) and controls (*P* = .0004). These findings suggest that the familial aggregation is, at least in part, explained by a polygenic effect of common low‐risk variants and rarer intermediate‐risk variants, while we did not find evidence of a role for novel recessive risk alleles.

## Abstract 2

**What's new?**

To find new breast cancer susceptibility alleles, these authors tested families in which at least three affected siblings had non‐BRCA1/2 breast cancer. No new susceptibility alleles emerged, but the analysis did reveal that on average, women from these families who had cancer had significantly higher polygenic risk scores than either sporadic cases or controls. This result highlights the importance of moderate risk alleles acting together in familial breast cancer.

**Abbreviations**
BCAC: Breast Cancer Association Consortium
BED: browser extensible data
CADD: combined annotation dependent depletion
GATK: genome analysis toolkit
GoNL: genome of the Netherlands
IBD: identical by descent
IGV: integrative genome viewer
LUMC: Leiden University Medical Center
NKI‐AvL: Netherlands Cancer Institute‐Antoni van Leeuwenhoek Ziekenhuis
OR: odds ratio
PRS: polygenic risk score
PTV: protein‐truncating variant
SNP: single nucleotide polymorphism
VUS: variant of uncertain significance

## 1 INTRODUCTION

Breast cancer is the most common cancer in females in the Western world and has a complex etiology in which both genetic and environmental factors affect disease risk. Having a family member affected by the disease is one of the most important risk factors.^1^ Pathogenic variants in the two most well‐known high‐risk breast cancer genes, *BRCA1* and *BRCA2*, explain approximately 17% of the familial relative risk.^2^ In addition, a number of less frequently mutated high‐risk genes (eg, *TP53*) and a number of genes in which pathogenic variants are associated with a more moderately increased risk (eg, *CHEK2*) together explain another 5%. Moreover, approximately 160 common polymorphisms have been associated with small increases in risk, which jointly explain about 18% of the excess familial risk.^3^

Since the discovery of *BRCA1* and *BRCA2*, several segregation studies have concluded that a polygenic model, or a model with a recessive allele would best explain the remaining familial risk.^4^, ^5^, ^6^, ^7^ Genetic searches for new loci, while successful, have focused on detecting rare dominant high‐risk alleles (by candidate gene re‐sequencing) or common low‐risk variants. Systematic searches for recessive alleles have not been conducted, despite evidence suggesting that such alleles could play a role in the genetic etiology of breast cancer. For example, a large meta‐analysis on familial breast cancer risk has shown that having a sister affected with breast cancer is associated with a stronger increase in risk than having a mother with breast cancer.^8^ In addition, an increased breast cancer risk has been reported in the offspring of consanguineous parents.^9^ Studies assessing regions of homozygosity in outbred populations have not shown more or larger regions of homozygosity in breast cancer cases, but some have suggested an increased frequency of homozygosity in specific genomic regions.^10^, ^11^

We performed a small‐scale search for recessive breast cancer risk alleles in families with at least three affected siblings and no other first or second‐degree relatives with early‐onset breast cancer. The regions in which all affected siblings shared two haplotypes, as determined by low‐density SNP arrays, were identified and used to filter the exome sequence data that was generated for one of the siblings. This approach significantly reduces the number of potentially interesting variants, allowing for less stringent filters on allele frequency and hence fewer assumptions about the characteristics of a novel breast cancer risk‐associated variant. In addition, we calculated a polygenic risk score based on 160 known breast cancer risk‐associated polymorphisms and assessed the contribution of exonic variants in known breast cancer susceptibility genes that were predicted to be damaging by in silico prediction algorithms.

## 2 METHODS

### 2.1 Selection of families

Families were ascertained through the clinical genetics centers of two Dutch hospitals, the Leiden University Medical Center (LUMC) and the Netherlands Cancer Institute Antoni van Leeuwenhoek Hospital (NKI‐AvL) and from a previously described set of breast cancer families collected throughout the Netherlands.^12^ We enriched for families with a presumed recessive mode of inheritance by selecting families in which at least three siblings were affected with breast cancer at any age. Sib‐ships that had first‐degree relatives with breast cancer in the previous or following generation were excluded, as were families with second‐degree relatives with breast cancer diagnosed before age 50. DNA from blood lymphocytes had to be available for at least two affected siblings. Availability of DNA samples from parents or other family members was not a selection criterion. In every family, at least one affected individual had been extensively tested according to local testing standards for pathogenic variants in *BRCA1* and *BRCA*2, and all families with a pathogenic variant or variant of uncertain significance in *BRCA1* or *BRCA2* were excluded.

### 2.2 Haplotype analysis

We genotyped all available DNA samples from the affected siblings using the HumanLinkage V Panel from Illumina. Sample preparation was done according to the manufacturer's protocol (Rev. B October 2010). Samples were hybridized to GoldenGate Universal‐32 BeadChip (Illumina) and chips were scanned using a Bead Array Reader (Illumina). The GenomeStudio software (version 2011.1, Illumina) was used to call genotypes. We used Merlin (v1.12) to calculate, for each sib pair and marker position, the probability that at this position the sib pair shared zero, one or two alleles identical by decent (IBD).^13^ On average a sib pair is expected to share two haplotypes in 25% of their genomes. To decrease the chance of false‐negative regions, we set a probability cut‐off such that for all sib pairs at least 25% of the markers were selected as sharing two alleles IBD (cut‐off: *P* > .05). We then selected all positions in which all siblings shared two alleles IBD or, for the analysis allowing for one phenocopy, all positions in which all but one sib shared two alleles IBD. These positions were converted into a BED file describing the regions IBD for both haplotypes. Each of these regions started one base pair after the last upstream position for which the affected siblings did not share two alleles IBD and ran until one base pair before the first downstream position for which they did not share two alleles IBD.

### 2.3 Exome sequencing and analysis

From each family, one affected individual was selected for exome sequencing of germline DNA. In most instances, this was the individual with the youngest age of diagnosis; however, in two families, another individual was selected due to limited availability of DNA. Samples were prepared using Illumina's Paired‐End Library Preparation Kit, after which the coding regions of the genome were captured using SeqCap EZ Exome v3.0 (Nimblegen). Sequencing was done on a HiSeq 2000 (Illumina), generating 2×100 base pair reads. We used GATK for indel realignment, base recalibration and finally variant calling using Haplotypecaller.^14^ These analyses were done according to the GATK best practices guidelines for DNA sequencing analysis. A detailed description of the settings and version numbers of the used software is given in Supporting Information.

### 2.4 Variant filtering and validation

**FIGURE 1:**
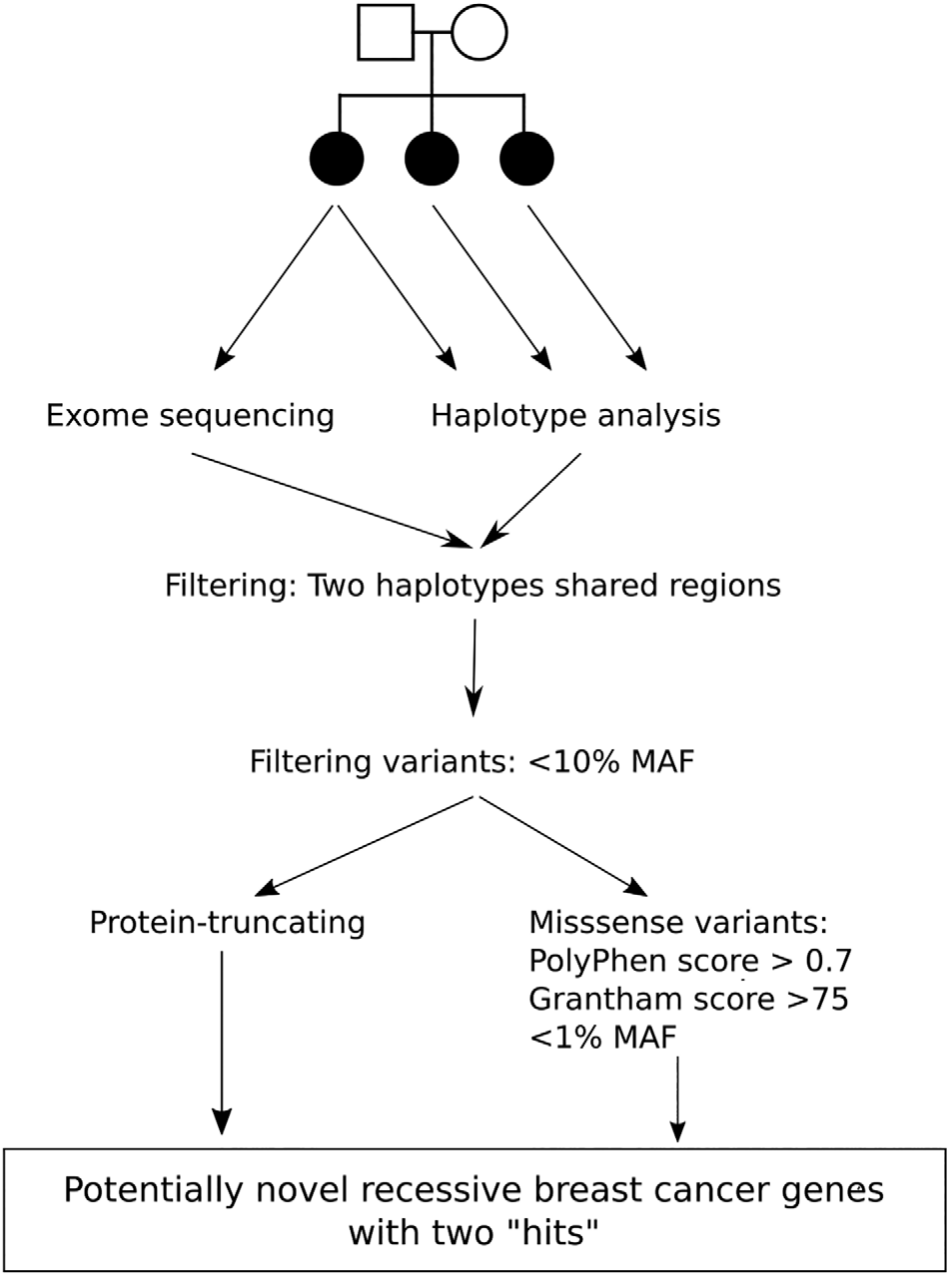
Strategy for the identification of recessively predisposing genetic variants. This overview presents our strategy for exome variant filtering to detect potential new breast cancer risk alleles with a recessive mode of inheritance

Figure 1 outlines our strategy for identifying recessively predisposing genetic variants in the affected sib ships. We first selected, for each individual, variants in regions in which they shared two haplotypes IBD with their siblings, using the family‐specific BED files. We then annotated the variants using Seattleseq (138, v9.03).^15^ Next, we selected all stop‐gained, frameshift and canonical splice site variants. These predicted protein‐truncating variants (PTV) could be either heterozygous or homozygous. We removed variants with an allele frequency >10% in either the exome variant server, Hapmap, 1000 genomes, ExAC or Genome of the Netherlands (GoNL) data.^16^, ^17^, ^18^, ^19^, ^20^ In addition, we removed all variants with an allele frequency of >30% in our dataset, since these are likely to be experiment‐specific artifacts. All remaining variants were manually inspected in the Integrative Genomics Viewer (IGV; v2.3.34) to remove any clear misalignments or other calling errors.^21^ In the genes in which a heterozygous potential PTV was found, we searched for a “second hit”, defined as either another potential PTV or a missense variant, satisfying the same frequency cut‐off. When two (or more) “hits” in a gene were identified, these variants were validated using Sanger sequencing. Primer sequences are available upon request.

We also considered a scenario in which two missense changes in a gene on two haplotypes could cause a recessive inheritance (ie, either homozygous or compound heterozygous). For this, we selected all missense changes in the regions specified by the BED files, with allele frequencies <1% and in silico annotations suggestive of deleteriousness (PolyPhen score > 0.7; Grantham score > 75).

### 2.5 Variants in known and suspected breast cancer genes

We examined a set of 35 known and suspected breast cancer susceptibility genes (derived from commercially available multigene panels, Table S1) for genetic variants regardless of haplotype sharing. The genes were assigned into four categories, based on the level of evidence for being associated with breast cancer risk (strong to unlikely); a separate category consisted of “syndromic” genes, in which variants have been associated with a range of cancers typical of certain familial cancer syndromes (*TP53*, *CDH1*, *PTEN*). PTVs in level 1/2 genes were filtered on allele frequency in the general population (exome variant server, Hapmap, 1000 genomes or GoNL) with a cut‐off of 0.1% for the high‐risk genes (*BRCA1*, *BRCA2*, *PALB2*, *TP53*, *PTEN* and *CDH1*) and 2% for the moderate risk genes (*ATM*, *CHEK2*), allowing for the observation that some PTVs in moderate‐risk genes (such as the c.1100delC in CHEK2) occur at >0.5% allele frequencies in some populations. All missense variants in the 35 genes were selected if their allele frequency in the general population was <2% and they had either a CADD^22^ score >20 or were found in one of the levels 1/2 genes. All selected variants were inspected manually in the IGV to remove misalignments. Variants that were both rare and not likely to result from a misalignment were then validated using Sanger sequencing.

### 2.6 Validation of potential recessive risk alleles

To further assess the association of selected variants with recessive breast cancer, we selected a set of 111 women diagnosed with breast cancer 35 or younger, through the clinical genetics center of the LUMC.

### 2.7 Polygenic risk score analysis

All affected sibs for whom DNA was available were genotyped using one of two SNP arrays partly designed to study SNPs associated with breast cancer risk: the iCOGs array and the OncoArray. To calculate polygenic risk scores, we selected all independent SNPs shown to be significantly (*P* < 5 × 10^−8^) associated with overall breast cancer by the Breast Cancer Association Consortium (BCAC), the largest case‐control study to date.^23^ The selected SNPs and respective ORs are shown in Table S2. A small number of known low‐risk variants were not included on the arrays. These variants were imputed with the help of IMPUTE2 based on the genome of the Netherlands (GoNL release 5.3) and 1000 genomes (Phase 3) data (Supporting Information).^18^, ^19^, ^24^ Polygenic risk scores were calculated using:

$${\mathrm{PRS}}_{j}=\sum_{i=1}^{160}n_{\mathrm{ij}}\;\ln\left({\text{OR}}_{i}\right)$$

where *n*_ij_ is the number of risk alleles (0, 1 or 2) SNP *i* carried by individual *j* and *OR*_*i*_ is the per‐allele odds ratio associated with SNP *i* (derived fromMichailidou et al^23^; Table S2). We compared the PRS of the family probands (the same individuals subjected to exome sequencing) with 357 sporadic cases and 327 age‐matched controls from the ORIGO study.^25^ These individuals were genotyped using the iCOGS array and imputed in the same way as the familial cases. The PRS was normalized based on the mean and SD of the ORIGO controls so that one unit in PRS corresponded to one SD. The odds ratio per unit SD of the PRS was obtained via univariate logistic regression within the ORIGO population. The null hypothesis of there not being a true difference in mean PRS between the “recessive” family probands, population cases and population controls was tested using a Welch two‐sample *t*‐test. All analyses were performed using R version 3.4.1.

All individuals provided informed consent and approval of the medical ethical committee at the LUMC was obtained.

## 3 RESULTS

### 3.1 Selected families and haplotype analysis

Nineteen families were selected for analysis (Figure S1). Samples were available from two affected siblings for three families, three affected siblings for 14 families and four affected siblings for two families. The average age at diagnosis of first primary breast cancer was 49.9. One family included a male breast cancer patient diagnosed at age 65. The “two haplotypes shared IBD” regions for each family covered on average 31.6%, 10.1% and 2.9% of the genome for families with two, three or four DNA samples available respectively. This is slightly higher than predicted proportions (25%, 6.25% and 1.6% respectively), but this was expected given our conservative IBD probability cut‐off (see Section 2).

### 3.2 Exome sequencing

Exome sequencing of one affected individual per family achieved 51× average on target coverage and detected on average 28 724 variants per individual. After filtering these variants based on the family‐specific haplotype sharing regions, an average of 10 775 (37.5%), 3222 (11.2%) and 734 (2.6%) variants remained in families with two, three or four individuals genotyped respectively. We first focused on variants that were predicted to result in a truncated protein. When a heterozygous protein‐truncating variant (PTV) was found, we assessed the gene for a second hit which could also be a missense variant (Table 1).

**TABLE 1:** rare protein‐truncating and missense variants found in the regions where the sibships share two haplotypes

Family	Gene	Variant (coding DNA)	Variant (protein)	Rs‐number	Co‐segregation^a^	Frequency in GoNL^b^ (%)
RF1	*PDIA2*	c.442C>T	p.R148*	rs370453080	2/3	0
		c.1418G>A	p.R473Q	rs116969376	3/3	1.3
RF4	*TLR5*	c.1174C>T	p.R392*	rs5744168	3/3	6.5
		c.541C>A	p.Q181K	rs45528236	3/3	6.5
RF6	*TRPM1*	c.4240G>T	p.E1414*	rs3784589	2/3	4.9
		c.1930G>A	p.V644M	rs17815774	3/3	4.7
RF13	*UNC93A*	c.625+1G>C	p.?	rs113906647	1/3	3.3
		c.1159T>C	p.Y387H	rs663227	1/3	0.7
RF14	*PLXNB3*	c.1629+2C>T	p.?	—	1/3	0
		c.4787T>A	p.V1596E	rs146832392	3/3	6.0
RF17	*CCHCR1*	c.121G>T	p.E41*	rs72856718	3/3	9.6
		c.2147G>A	p.R716Q	rs130072	3/3	9.6
		c.803T>A	p.L268Q	rs11540822	3/3	9.6

a Indicates the number of siblings carrying the allele out of the total number of siblings from this family tested.

b Frequency in Genome of the Netherlands: genome sequences of 998 independent Dutch individuals.^22^ Accession numbers for the transcripts and protein sequences used to describe the variants: *PDIA2*: NM_006849.2, NP_006840.2; *TLR5*: NM_003268.5, NP_003259.2; *TRPM*: NM_001252020.1, NP_001238949.1; *UNC93A*: NM_018974.3, NP_061847.2; *PLXNB3*: NM_005393.2, NP_005384.2; *CCHCR1*: NM_001105564.1, NP_001099034.1.

We originally set the PTV allele frequency cut‐off relatively high (<10%) to allow for the possibility of a single variant that was homozygous in multiple families. No such variants were detected in our dataset, but we did find six genes with two or more heterozygous positions in six different families. For compound heterozygotes, we assumed that the allele frequency of a potentially causal variant was lower (<2%), rendering the variants in *TLR5*, *TRPM1*, *UNC93A*, *PLXNB3* and *CCHCR1* unlikely candidates. In the remaining gene, *PDIA2*, we identified a PTV p.R148* and a missense variant p.R473Q, shared IBD in one family. *PDIA2* encodes an oxidoreductase involved in protein folding and specifically expressed in the pancreas.^26^, ^27^, ^28^ In addition, it binds estrogen (specifically 17β‐estradiol) and might buffer the local estrogen levels in the pancreas.^29^ To further examine the possibility that variants in *PDIA2* are associated with breast cancer, we genotyped a set of 111 patients diagnosed with breast cancer before the age of 35 for the two variants detected in family RF1. The PTV p.R148* was not observed, while the missense variant p.R473Q was detected twice (0.9%). The allele frequency of 1.3% in the Genome of the Netherlands, also suggests that this variant is not associated with breast cancer.^19^

A similar filter for missense variants revealed two rare homozygous missense variants, *SERINC2* p.R126W in family RF4 and *ZNF717* p.H63L in family RF7 (Table S3).

*SERINC2* regulates lipid biosynthesis and incorporates serine into membrane lipids, while the function of *ZNF71* is unknown. The CADD scores for both variants were <20. Based on this, neither variant was considered as a serious candidate for follow‐up studies.

### 3.3 Analyses allowing for one phenocopy

Since breast cancer is a common disease, there is a high probability that a case in a family is not genetic (ie, a phenocopy). Therefore, we assessed the regions of the genome where only two out of three (or three out of four) affected sisters share two haplotypes. PTVs obtained in this way were then filtered as in the previous analysis (Table 2). Again, most variants were relatively common, but did not occur in multiple families. The only gene in which variants are rare enough to be a possible candidate was *SLC26A10*, with variants c.1206G>A (p.W402*) and c.1247T>G (p.L416R) found in family RF2. Both variants were shared by two of the three affected sisters. However, in GoNL, both variants were present in the same seven individuals and predicted to be on the same haplotype, excluding the possibility of compound heterozygosity. *SCL26A10* has no known function and has been suggested to be an imprinted, maternally expressed, pseudogene.^30^, ^31^

**TABLE 2:** Rare protein‐truncating and missense variants found in the regions where the sibships share two haplotypes, allowing for one phenocopy

Family	Gene	Variant (coding DNA)	Variant (protein)	Rs‐number	Co‐segregation^a^	Frequency in GoNL^b^ (%)
RF2	*ZAN*	c.1249 + 1G>A	p.?	rs117406702	3/3	3.8
		c.8132C>T	p.P2711L	rs201771583	3/3	0
	*SLC26A10*	c.1206G>A	p.W402*	rs113207856	2/3	0.7
		c.1247T>G	p.L416R	rs111924104	2/3	0.7
RF6	*CCHCR1*	c.121G>T	p.E41*	rs72856718	1/3	9.6
		c.803T>C	p.L232Q	rs11540822	1/3	9.6
RF8	*PLA2G4C*	c.893delC	p.P298fs	rs11564598	3/3	2.9
		c.452C>T	p.P151L	rs11564538	1/3	5.0
RF14	*PKHD1L1*	c.7246 + 1G>C	p.?	rs17368310	3/3	4.5
		c.10310A>G	p.D3437G	rs118053060	2/3	2.5

a Indicates the number of siblings carrying the allele out of the total number of siblings from this family tested.

b Frequency in Genome of the Netherlands: genome sequences of 998 independent Dutch individuals.^22^ Accession numbers for the transcripts and protein sequences used to describe the variants: *ZAN*: NM_003386.2, NP_003377.2; *SLC26A10*: NM_133489.2, NP_597996.2; *CCHCR1*: NM_001105564.1, NP_001099034.1; *PLA2G4C*, NM_003706.2, NP_003697.2; *PKHD1L1*: NM_177531.4, NP_803875.2.

### 3.4 Known and suspected moderate and high‐risk genes

We next examined 35 genes in which PTVs have been demonstrated or suspected to be associated with breast cancer risk (Tables 3 and S1). We found two rare missense variants in known high‐risk genes, one in *PALB2* and one in *BRCA2*. ClinVar lists the variant in *PALB2* as benign, the one in *BRCA2* as variant of uncertain significance (VUS). Family RF17 was included in our study as being non‐*BRCA1/2* because the sister not carrying the missense variant was the one tested in the clinical setting. No studies on the functional effects of this variant have been published to date, but the CADD score of 35 indicates that it might affect protein function. Therefore, it is possible that this family harbors a pathogenic *BRCA2* variant.

**TABLE 3:** Rare genetic variant in known and suspected breast cancer genes

Gene	Family	Variant (coding DNA)	Variant (protein)	Rs‐number	Co‐segregation^a^	Frequency^b^ (%)
*ATM*	RF6	c.146C>G	p.S49C	rs1800054	2/3	1.7
*ATM*	RF7	c.2531G>A	p.G844E	rs587781808	2/3	0.002
*ATM*	RF10	c.2991A>G	p.(=)	rs1203368496	3/3	0
*ATM*	RF18	c.584C>T	p.T195I	rs1196611507	2/3	—
*ATM*	RF20	c.146C>G	p.S49C	rs1800054	3/3	1.7
*BRCA2*	RF17	c.8290G>A	p.A2764T	rs786202189	2/3	—
*CDH1*	RF21	c.1689C>T	p.(=)	rs587780786	2/2	0.007
*CHEK2*	RF4	c.1100delC	p.T367fs	rs555607708	3/3	1
*CHEK2*	RF8	c.1100delC	p.T367fs	rs555607708	3/3	1
*CHEK2*	RF14	c.556A>C	p.N186H	rs146198085	1/3	0.01
*PALB2*	RF20	c.150A>T	p.K50N	—	1/2	–
*RAD51C*	RF8	c.790G>A	p.G264S	rs147241704	3/3	0.3
*RAD51C*	RF19	c.790G>A	p.G264S	rs147241704	1/2	0.3

a Indicates the number of siblings carrying the allele out of the total number of siblings from this family tested.

b Highest frequency in either ESP, ExAc, gnomAD, or GoNL; — if no entry listed; Accession numbers for the transcripts and protein sequences used to describe the variants: *ATM*: NM_000051.3, NP_000042.3; *BRCA1*: NM_007294.3, NP_009225.1; *BRCA2*: NM_000059.3, NP_000050.2; *CDH1*: NM_004360.3, NP_004351.1; *CHEK2*: NM_007194.3, NP_009125.1; *PALB2*: NM_024675.3, NP_078951.2; *RAD51C*: NM_058216.2, NP_478123.1.

The c.1100delC pathogenic variant in *CHEK2*, associated with an odds ratio (OR) of approximately 2.3,^32^ was found in all affected individuals of families RF4 and RF8, with all individuals being heterozygous. We found several missense variants in the (suspected) moderate‐risk genes *ATM*, *CHEK2* and *RAD51C*. The effect of missense changes in *ATM* and *CHEK2* on breast cancer risk is, besides a few specific examples, largely uncertain.^33^, ^34^, ^35^ None of the variants listed in Table 3 belong to any of these exceptions, but some do have CADD scores >20 suggestive of pathogenicity. Two other variants have previously been associated with breast cancer risk, although association data have been conflicting. *ATM* c.146C>G (p.S49C) was detected in families RF6 and RF20; its associated breast cancer risk is unlikely to be larger than 1.5.^34^, ^36^, ^37^ Likewise, conflicting results were obtained for the breast and/or ovarian cancer risk of *RAD51C* c.790G>A (p.G264S) in families RF8 and RF19.^38^ The contribution of these variants to breast cancer susceptibility, if any, is therefore uncertain.

### 3.5 Polygenic risk score analysis

**FIGURE 2:**
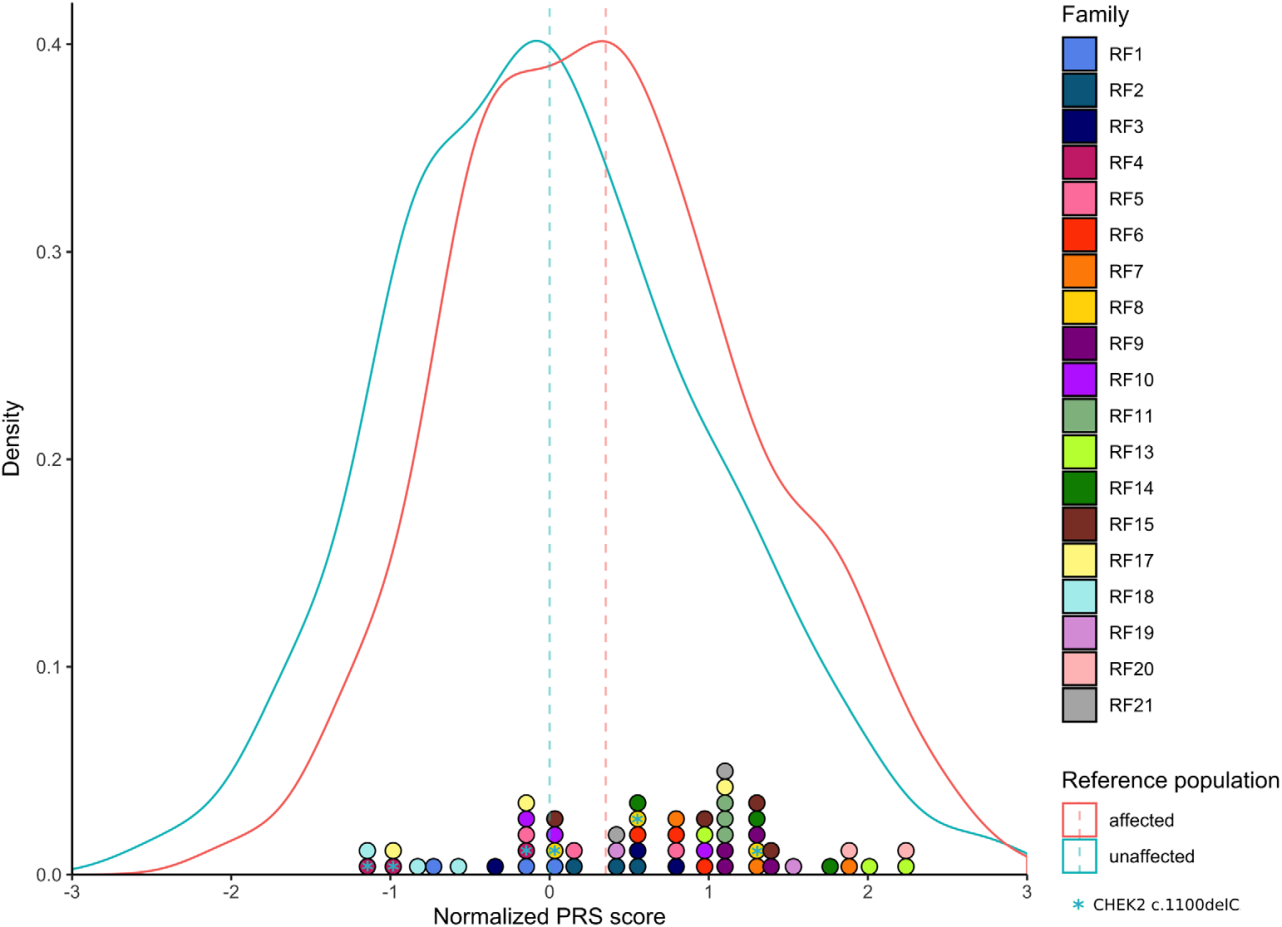
PRS scores for recessive families compared to population cases and controls. The blue and red line represent the density plots of PRS for population controls and cases, respectively. Colored circles at the ordinate each represent one individual from the 19 investigated families, circles with the same color belong to the same family. Circles with a blue star represent carriers of the CHEK2 c.1100delC variant. The dotted lines represent the mean PRS for the population controls and familial cases [Color figure can be viewed at wileyonlinelibrary.com]

Over 160 independent common SNPs have been found to be convincingly associated with breast cancer and can be combined into a PRS^23^(Table S2). To examine the effect of the PRS on the breast cancer cases in our families, we genotyped or imputed these SNPs for all individuals from whom DNA was available. The PRS was normalized such that the mean and SD of the population controls were 0 and 1, respectively. Figure 2 shows the difference in distribution between our familial cases and a set of population cases and controls, clearly showing a strong skewing toward PRS >0 for the familial cases. The odds ratio per unit SD of the PRS was 1.46. The average PRS of all the affected siblings in the families was 0.63, corresponding to an odds ratio (OR) of 1.27. The average score of the family probands (0.81, OR 1.36) was significantly higher than that in both population cases (0.35, OR 1.14, *P* = .026) and controls (*P* = .0004).

## 4 DISCUSSION

In our study, we assessed whether breast cancer in families with at least three affected siblings, can be explained by a susceptibility gene with a recessive mode of inheritance. After a haplotype‐guided exome analysis, we identified no homozygous or compound‐heterozygous variants that were likely to explain the clustering of breast cancers in the selected families. We did identify two families in which all affected individuals carry the known moderate risk variant CHEK2*1100delC. Furthermore, we showed that on average, the affected women in these families had significantly higher PRS than both sporadic cases and population controls. Together, these results indicate that our selection criteria enrich for these factors and suggest that, rather than being caused by a single highly penetrant variant, increased breast cancer risk in some of these families may be due to the combined effect of multiple rare and common genetic variants with varying effect‐sizes, and perhaps other nongenetic risk factors as well.

Due to a few limitations of our study, we cannot completely rule out that some of our families are nonetheless explained by recessive risk alleles. First, some of the variants we identified (eg, *PDIA2* p.R148*) are so rare in the general population that they would require very large case‐control populations to assess their association with breast cancer. As they grow in size, publicly available reference datasets and databases in which variants in potential disease‐associated genes can be reported are becoming very valuable for this purpose. Second, a recessive risk allele might be located outside the protein‐coding regions of the genome and thus not be captured by an exome sequencing approach. Moreover, structural variation, affecting more than a few base pairs, is mostly undetectable with the methods used in our study. Whole‐genome sequencing would identify these, but their mostly poor genomic annotation will make their filtering for follow‐up analyses very hard.

Third, our family selection has led to many sibships that could also be explained by a dominant allele with incomplete penetrance. While our study design had advantages for the variant filtering, there are alternative ways to enrich for recessive alleles, such as population‐based sib pairs or early‐onset cases with unaffected parents. Such studies have not yet been published for breast cancer but would probably also suffer from severe genetic model heterogeneity. Thus, the existence of recessive breast cancer alleles remains possible, although it is remarkable in this regard, that only a handful of the >160 common breast cancer loci derived from population‐based genome‐wide association studies affect risk in a recessive mode, rather than in a co‐dominant way.^25^

Nonetheless, our results are in agreement with previous exome sequencing studies in non‐*BRCA1/2* familial breast cancer cases. Although more than 20 such studies have been published, only two new breast cancer genes suggested by these studies were replicated independently: *FANCM* and *RECQL*.^39^, ^40^, ^41^, ^42^ Most of these studies, however, reported pathogenic variants in known moderate‐risk genes. Studies employing gene panel sequencing in a large numbers of familial breast cancer cases suggest that approximately 4% carry a pathogenic or likely pathogenic variant in a breast cancer gene other than *BRCA1* or *BRCA2*.^43^, ^44^, ^45^ We found two index cases carrying the CHEK2*1100delC pathogenic variant (consistent with high frequency of this variant in the Dutch population), and four possibly pathogenic variants in other susceptibility genes. At least for CHEK2*1100delC it has been shown that the risk associated with this pathogenic variant and the risk associated with a PRS combine multiplicatively.^46^ With regard to the common low‐risk variants, our results are consistent with studies which have found that non‐*BRCA1/2* familial breast cancer cases have a higher PRS than both cases from the general population and cases who carry a *BRCA1* or *BRCA2* pathogenic variant.^47^, ^48^, ^49^, ^50^ Whether the prevalence of rare missense variants in the known breast cancer genes we observed in our families is causally linked to breast cancer, will need very large case‐control studies to substantiate further.

The enrichment of moderate and low‐risk alleles among the cases of at least part of the families in our study adds to a growing body of evidence on the importance of this type of risk alleles in causing familial breast cancer. Multigene panel sequencing has rendered the detection of rare variation in known risk genes standard clinical genetic practice, but the genotyping of the many common low‐risk alleles is not yet routinely performed in this setting. Nonetheless, the risks associated with the PRS and the likely multiplicative way in which it combines with those of pathogenic variants in moderate‐risk genes argue for a more comprehensive approach to genetic testing and counseling. This calls for the development of integrative risk prediction models, including the effect of mammographic density, lifestyle and environmental risk factors.

## CONFLICT OF INTEREST

The authors declare that they have no conflicts of interest.

## ETHICS STATEMENT

All individuals provided informed consent and approval of the medical ethical committee at the LUMC was obtained (protocols P49/99 and P09.203).

## Supporting information


**Data S1** Supporting Information.

## Data Availability

The data that support the findings of our study are available on request from the corresponding author. The data are not publicly available due to privacy or ethical restrictions.
