# Supplementary material for: Clustering of known low and moderate risk alleles rather than a novel recessive high‐risk gene in non‐BRCA1/2 sib trios affected with breast cancer
Source: Int J Cancer. 2020 May 30;147(10):2708–16. doi: 10.1002/ijc.33039 (PMC7540545; doi:10.1002/ijc.33039)
Supplement: Supplementary file 1 — Data S1 Supporting Information. [file IJC-147-2708-s001.pdf]

Clustering of known low and moderate risk alleles rather than a novel recessive high-risk gene in non-BRCA1/2 sib trios affected with breast cancer

Florentine S. Hilbers, Peter J. van 't Hof, Caro M. Meijers, Hailiang Mei, Kyriaki Michailidou, Joe Dennis, Frans B. L. Hogervorst, Petra M. Nederlof, Christi J. van Asperen, Peter Devilee

Table of content

Supplementary Methods	2
Supplementary Table 1: Classification of genes according to level of evidence that protein-truncating variants in these genes are associated with breast cancer	3
Supplementary Table 2: Overview of the SNPs included in the polygenic risk score	4
Supplementary Table 3: Rare missense variants found in the regions where the sibships share two haplotypes	8
Supplementary Figure 1: Pedigrees of the families included in this study	9
References	12

Supplementary Methods

Exome sequencing read alignment and variant calling

Analysis of the sequence data was performed within the in-house pipeline framework Biopet (version 0.1.3)[1]. First, raw reads were trimmed based on quality using Sickle (version 1.200)[2] and adapter sequences were removed with the help of Cutadapt (version 1.1)[3]. Afterwards, the quality of the reads was assessed using Fastqc (version 0.10.1)[4]. The reads were then aligned to the human reference genome (hg19) with the help of BWA (version 0.7.8-r455)[5]. After alignment Picard (version 1.109.1722)[6] was used to sort the bam files and to mark duplicate reads. Using GATK (version 3.1-1-gcfc45fd)[7] we applied base quality score recalibration, indel realignment, called variants using HaplotypeCaller and recalibrated variant quality scores using Hapmap, Omni, 1000G and dbSNP for single nucleotide variants and Mills and dbSNP for indels (datasets as provided in gatk_bundle_2.5). Next, variants in the regions described in the family-specific BED files were selected using vcftools (v1.12b)[8].

Imputation of SNP array data for PRS calculation

Imputation was performed (without pre-phasing) with IMPUTE2 (version 2.3.2)[9] using both 1000G (phase 3 b37 haplotypes)[10] and GoNL (release5.3, imputation ready haplotypes)[11] as a reference. We imputed a region of 1Mb around every SNP of interest with a buffer of 500 kb. We set “k” to 200 and “k_hap” to 2000 and 998, for 1000G and GoNL respectively. Before imputation the reference panels were merged” using the “merge_ref_panels” option. The “effective size" of the population (Ne) was set to 20000. To replicate this analysis use “seed” 8256245.

Supplementary Figure 1. Pedigrees of the families included in this study

H indicates that an individual’s germline DNA was haplotyped, HE indicates that an individual’s germline DNA was both haplotyped and exome sequenced. Under each affected individual for whom germline DNA was available the normalized PRS and OR (in italics) are indicated. The * -symbol indicates individuals carrying the CHEK2*1100delC variant.
B= breast cancer, Bl= bladder cancer, Br= brain cancer, C= colon cancer, Ca= cancer not otherwise specified, Cx= cervical cancer, E= esophagus cancer, Hy= hypophysis cancer, L= lung cancer, Leu= leukemia, Li= liver cancer, Ly= lymphoma (not specified), M= melanoma, NHL= non-Hodgkin lymphoma, P= Prostate cancer, Pa= pancreas cancer, Re= renal cancer, S= stomach cancer, Sk = skin cancer (not specified), T= testicular cancer, Th= thyroid cancer.

Supplementary Table 1

Classification of genes according to level of evidence that protein-truncating variants in these genes are associated with breast cancer

Evidence level*	Genes
1. Strong	ATM, BRCA1, BRCA2, CHEK2, PALB2
2. Syndromic	CDH1, PTEN, TP53
3. Likely	BARD1, BRIP1, FANCC, FANCM, NF1, RAD51C, RAD51D
4. Suggestive	AKT1, MEN1, MSH6, NBN, PIK3CA, RECQL, STK11
5. Unlikely	ATR, EPCAM, FAM175A, GEN1, MLH1, MRE11A, MSH2, MUTYH, PMS2, PPM1D, RAD50, RINT1, XRCC2

* 1: association has been demonstrated in multiple publications; 2: familial cancer syndromes in which breast cancer is a linked feature; 3: some controversy among studies, but meta-analyses positive; 4: a few positive studies, but no firm replication yet; 5: anecdotal evidence, or studies controversial

Supplementary Table 2
Overview of the SNPs included in the polygenic risk score

SNP	Ref	Alt	OR*	Beta**
rs616488	A	G	0.94	-0.06
rs2992756	T	C	0.94	-0.06
rs4233486	C	T	1.03	0.03
rs79724016	T	G	0.93	-0.07
rs1707302	A	G	1.04	0.04
rs140850326	Del	Ins	0.97	-0.03
rs17426269	G	A	1.05	0.05
rs12022378	C	T	1.04	0.04
rs7529522	T	C	1.06	0.06
rs11249433	A	G	1.11	0.10
rs12405132	C	T	0.97	-0.03
rs12048493	A	C	1.04	0.04
rs4971059	G	A	1.05	0.05
rs35383942	C	T	1.12	0.11
rs11117758	G	A	0.95	-0.05
rs72755295	A	G	1.15	0.14
rs113577745	C	G	1.08	0.08
rs12710696	T	C	0.97	-0.03
rs6725517	A	G	0.96	-0.04
rs3833441	Del	Ins	1.09	0.09
rs4849887	T	C	1.10	0.09
rs2016394	G	A	0.95	-0.05
rs1550623	G	A	1.05	0.05
rs1830298	C	T	0.94	-0.06
rs34005590	C	A	0.82	-0.20
rs4442975	G	T	0.89	-0.12
rs16857609	C	T	1.06	0.06
rs12479355	A	G	0.96	-0.04
rs6762644	A	G	1.05	0.05
rs4973768	C	T	1.11	0.10
rs12493607	G	C	1.05	0.05
rs6796502	G	A	0.92	-0.08
rs1053338	A	G	1.05	0.05
rs6805189	T	C	0.97	-0.03
rs13066793	A	G	0.94	-0.06
rs9833888	G	T	1.06	0.06
rs34207738	Del	Ins	1.06	0.06
rs58058861	G	A	1.06	0.06

rs6815814	A	C	1.06	0.06
rs10718573	Ins	Del	0.96	-0.04
rs10022462	C	T	1.04	0.04
rs9790517	C	T	1.04	0.04
rs77528541	G	T	0.95	-0.05
rs6828523	C	A	0.91	-0.09
rs116095464	T	C	1.06	0.06
rs3215401	Del	Ins	0.93	-0.07
rs10069690	C	T	1.06	0.06
rs2012709	C	T	1.02	0.02
rs10941679	A	G	1.15	0.14
rs62355902	A	T	1.18	0.17
rs10472076	T	C	1.03	0.03
rs1353747	T	G	0.96	-0.04
rs72749841	T	C	0.93	-0.07
rs35951924	Del	Ins	0.95	-0.05
rs7707921	T	A	1.06	0.06
rs10474352	C	T	0.94	-0.06
rs6882649	G	T	1.03	0.03
rs6596100	C	T	0.94	-0.06
rs1432679	C	T	0.93	-0.08
rs4562056	G	T	1.05	0.05
rs204247	G	A	0.96	-0.04
rs3819405	C	T	0.96	-0.04
rs2223621	T	C	0.96	-0.04
rs71557345	G	A	0.92	-0.08
rs17529111	T	C	1.02	0.02
rs12207986	G	A	1.03	0.03
rs3757322	T	G	1.08	0.08
rs9397437	G	A	1.17	0.16
rs2747652	T	C	1.06	0.06
rs6569648	C	T	1.06	0.06
rs7971	A	G	0.96	-0.04
rs17156577	T	C	1.05	0.05
rs6964587	G	T	1.03	0.03
rs17268829	T	C	1.05	0.05
rs71559437	G	A	0.93	-0.07
rs4593472	C	T	0.97	-0.03
rs11977670	G	A	1.06	0.06
rs720475	G	A	0.96	-0.04
rs9693444	A	C	0.94	-0.06
rs13365225	A	G	0.91	-0.09

rs6472903	G	T	1.06	0.06
rs2943559	A	G	1.10	0.10
rs514192	A	T	0.95	-0.05
rs12546444	A	T	0.93	-0.07
rs13267382	A	G	0.97	-0.03
rs58847541	G	A	1.08	0.08
rs13281615	A	G	1.11	0.10
rs11780156	C	T	1.05	0.05
rs1011970	G	T	1.07	0.07
rs10759243	C	A	1.06	0.06
rs676256	C	T	1.10	0.09
rs10816625	A	G	1.11	0.10
rs13294895	C	T	1.06	0.06
rs1895062	A	G	0.94	-0.06
rs10760444	G	A	0.97	-0.03
chr9:136151579	Ins	Del	1.03	0.03
rs67958007	Ins	Del	1.09	0.09
rs7072776	A	G	0.95	-0.05
rs11814448	A	C	1.12	0.11
rs10995201	A	G	0.90	-0.11
rs704010	T	C	0.93	-0.07
rs140936696	Ins	Del	0.96	-0.04
rs7904519	A	G	1.03	0.03
rs11199914	C	T	0.96	-0.04
rs35054928	Ins	Del	0.79	-0.24
rs45631563	A	T	1.23	0.21
rs2981578	C	T	0.81	-0.21
rs3817198	T	C	1.05	0.05
rs6597981	A	G	1.04	0.04
rs3903072	G	T	0.97	-0.03
rs554219	C	G	1.21	0.19
chr11:69088342	C	A	1.28	0.25
rs11820646	T	C	1.04	0.04
rs12422552	G	C	1.06	0.06
rs7297051	C	T	0.89	-0.12
rs202049448	T	C	0.95	-0.05
rs17356907	A	G	0.91	-0.09
rs1292011	A	G	0.92	-0.08
rs206966	C	T	1.05	0.05
rs11571833	A	T	1.35	0.30
rs6562760	A	G	1.05	0.05
rs2236007	G	A	0.93	-0.07

rs2588809	T	C	0.94	-0.06
rs999737	C	T	0.91	-0.09
rs941764	A	G	1.03	0.03
rs11627032	T	C	0.96	-0.04
rs10623258	Del	Ins	1.04	0.04
rs2290203	G	A	0.94	-0.06
rs4784227	C	T	1.23	0.21
rs17817449	T	G	0.95	-0.05
rs11075995	A	T	0.97	-0.03
rs28539243	G	A	1.05	0.05
rs2432539	A	G	0.97	-0.03
rs13329835	A	G	1.07	0.07
rs4496150	C	A	0.96	-0.04
rs146699004	Ins	Del	0.97	-0.03
rs72826962	C	T	1.20	0.18
chr17:44252468	G	A	0.95	-0.05
rs2787486	A	C	0.93	-0.07
rs745570	A	G	1.05	0.05
rs527616	C	G	1.03	0.03
rs1436904	T	G	0.95	-0.05
rs117618124	T	C	0.89	-0.12
rs6507583	A	G	0.92	-0.08
rs78269692	T	C	1.09	0.09
rs2594714	G	A	0.97	-0.03
rs2965183	G	A	1.04	0.04
chr19:17262404	C	G	1.03	0.03
rs4808801	A	G	0.93	-0.07
rs71338792	Del	Ins	1.05	0.05
rs3760982	A	G	0.95	-0.05
rs16991615	G	A	1.10	0.10
rs6122906	A	G	1.05	0.05
rs2823093	G	A	0.94	-0.06
rs17879961	A	G	1.26	0.23
rs132390	C	T	0.96	-0.04
rs6001930	T	C	1.12	0.11
rs738321	C	G	0.95	-0.05
rs73161324	C	T	1.06	0.06
rs28512361	G	A	1.05	0.05

*odds ratios (OR) for the alternative (Alt) allele derived from Michailidou et al. 2017[12], ** beta for the Alt allele calculated based on the ORs from Michailidou et al. 2017[12]

Supplementary Table 3
Rare missense variants found in the regions where the sibships share two haplotypes

Gene	Family	Variant (coding DNA)	Variant (protein)	Rs-number	Frequency*	CADD
SERINC2	RF4	c. 364C>T	p.R126W	rs183001614	0.0053	19.010
ZNF717	RF7	c.188T>A	p.H63L	rs201105907	0.0001	0.015

* Frequency in GnomAD[13] Accession numbers for the transcripts and protein sequences used to describe the variants can be found in Supplementary table 1

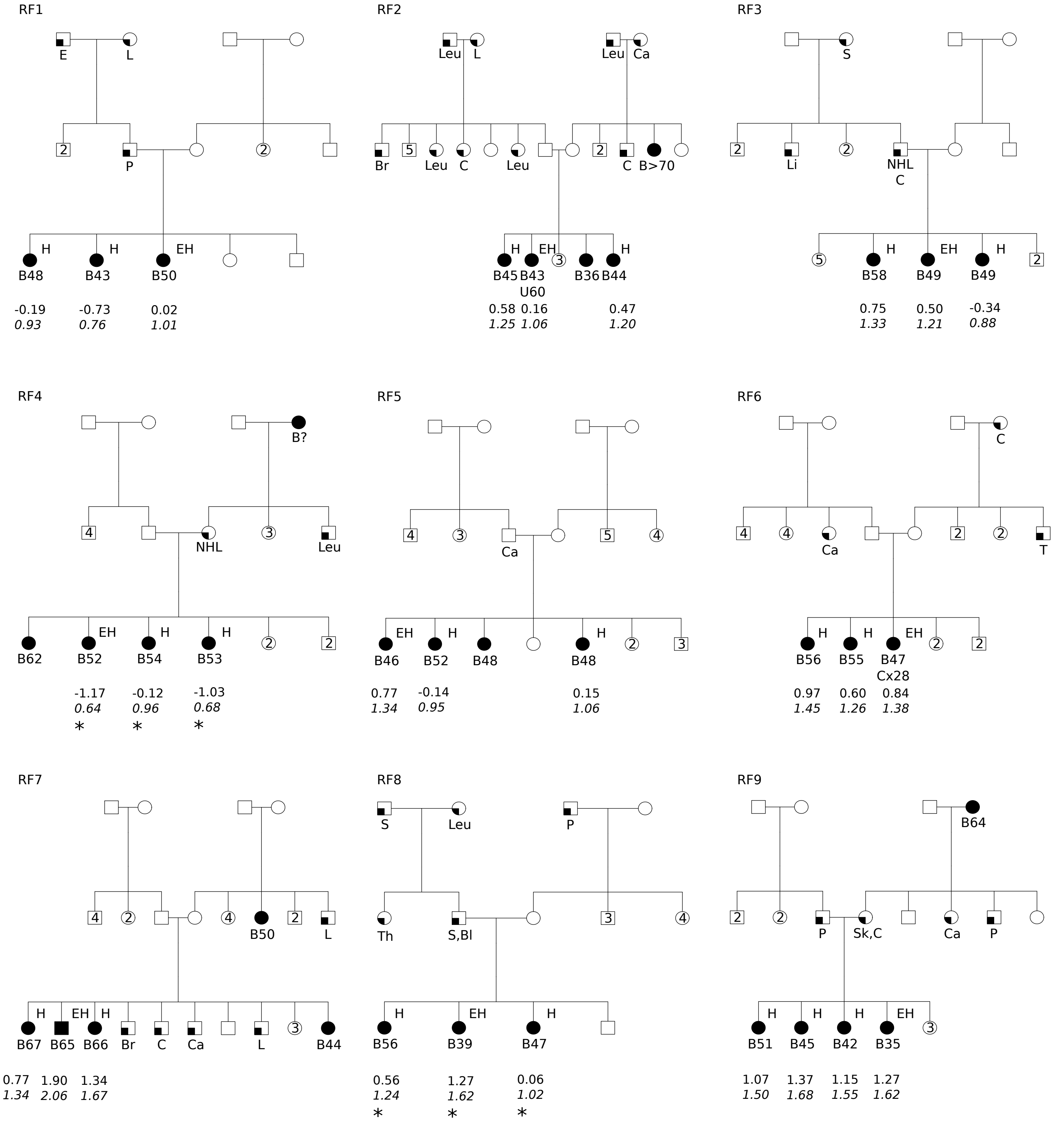
RF1
E
L
2
P
2
H
H
EH
B48
B43
B50
-0.19
0.93
-0.73
0.76
0.02
1.01
RF2
Leu
L
Leu
Ca
Br
5
Leu
C
Leu
2
C
B>70
H
EH
3
H
B45
B43
U60
B36
B44
0.58
1.25
0.16
1.06
0.47
1.20
RF3
S
2
Li
2
NHL
C
5
H
EH
H
2
B58
B49
B49
0.75
1.33
0.50
1.21
-0.34
0.88
RF4
B?
4
NHL
3
Leu
EH
H
H
2
2
B62
B52
B54
B53
-1.17
0.64
*
-0.12
0.96
*
-1.03
0.68
*
RF5
4
3
Ca
5
4
EH
H
H
2
3
B46
B52
B48
B48
0.77
1.34
-0.14
0.95
0.15
1.06
RF6
C
4
4
Ca
2
2
T
H
H
EH
2
2
B56
B55
B47
Cx28
0.97
1.45
0.60
1.26
0.84
1.38
RF7
4
2
4
B50
2
L
H
EH
H
3
B67
B65
B66
Br
C
Ca
L
B44
0.77
1.34
1.90
2.06
1.34
1.67
RF8
S
Leu
P
Th
S,Bl
3
4
H
EH
H
B56
B39
B47
0.56
1.24
*
1.27
1.62
*
0.06
1.02
*
RF9
B64
2
2
P
Sk,C
Ca
P
H
H
H
EH
3
B51
B45
B42
B35
1.07
1.50
1.37
1.68
1.15
1.55
1.27
1.62

RF10

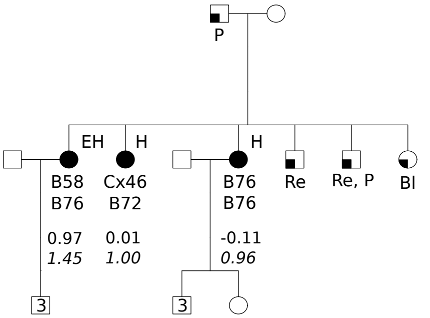

RF11

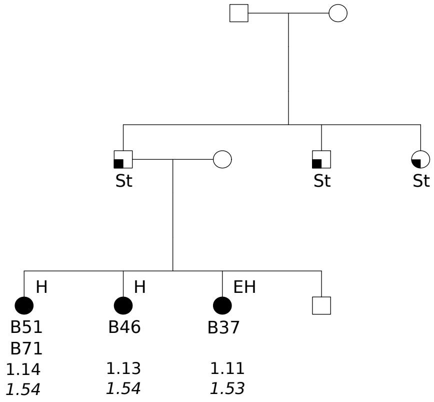

RF13

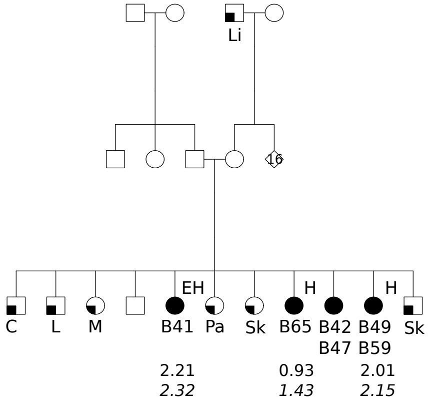

RF14

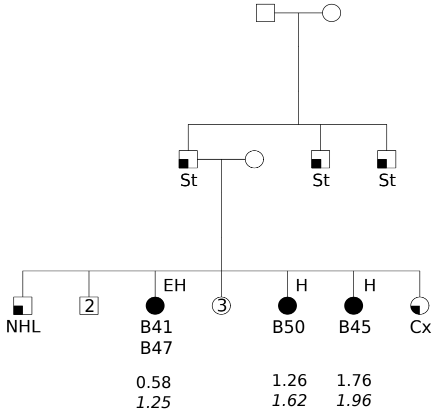

RF15

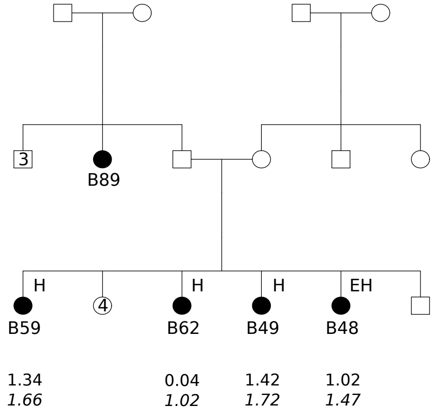

RF17

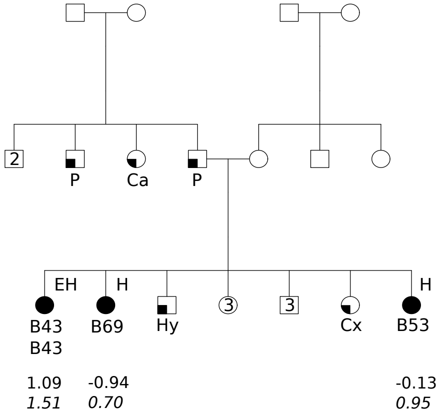

RF18

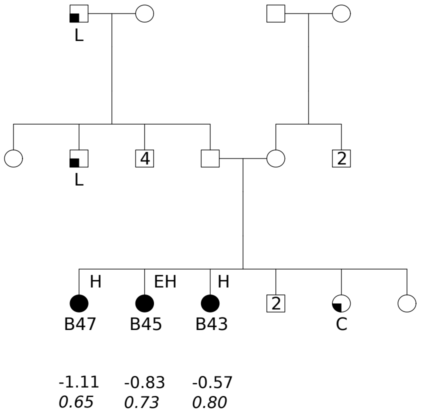

RF19

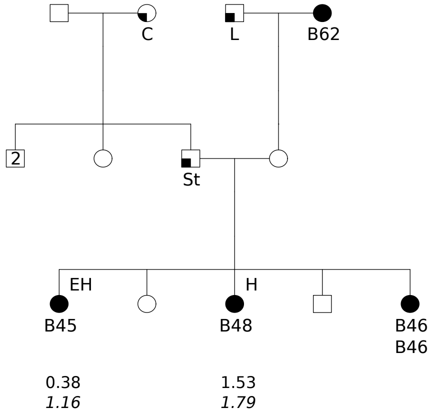

RF20

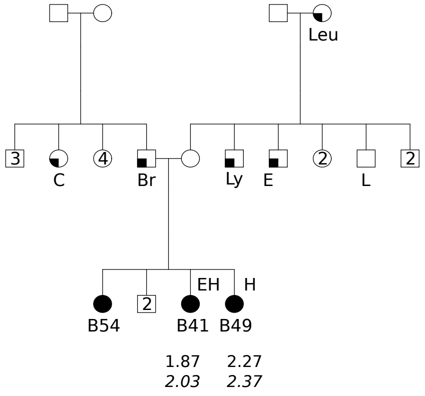

RF21

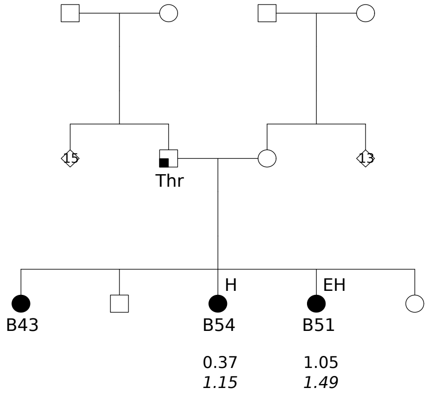

Supplementary Figure 1. Pedigrees of the families included in this studyH indicates that an individual’s germline DNA was haplotyped, EH indicates that an individual’s germline DNA was both haplotyped and exome sequenced. Under each affected individual for whom germline DNA was available the normalized PRS and OR (in italics) are indicated. The * -symbol indicates individuals carrying the CHEK2*1100delC variant.B= breast cancer, Bl= bladder cancer, Br= brain cancer, C= colon cancer, Ca= cancer not otherwise specified, Cx= cervical cancer, E= esophagus cancer, Hy= hypophysis cancer, L= lung cancer, Leu= leukemia, Li= liver cancer, Ly= lymphoma (not specified), M= melanoma, NHL= non-Hodgkin lymphoma, P= Prostate cancer, Pa= pancreas cancer, Re= renal cancer, S= stomach cancer, Sk = skin cancer (not specified), T= testicular cancer, Th= thyroid cancer.

References

1 The Leiden University Medical Centre. *Biopet. (0.1.3)*. 2014.

2 Joshi N, Fass J. Sickle: A sliding-window, adaptive, quality-based trimming tool for FastQ files. Published Online First: 2011.http://github.com/najoshi/sickle (accessed 14 Apr2017).

3 Martin M. Cutadapt removes adapter sequences from high-throughput sequencing reads. *EMBnet.journal* 2011;**17**:10–2.

4 Babraham Institute. *Fastqc. (0.10.1)*. 2012.

5 Li H, Durbin R. Fast and accurate short read alignment with Burrows-Wheeler transform. *Bioinforma Oxf Engl* 2009;**25**:1754–60.

6 Broad Institute. *Picard. (1.109.1722)*. 2014.

7 McKenna A, Hanna M, Banks E, Sivachenko A, Cibulskis K, Kernytsky A, Garimella K, Altshuler D, Gabriel S, Daly M, DePristo MA. The Genome Analysis Toolkit: a MapReduce framework for analyzing next-generation DNA sequencing data. *Genome Res* 2010;**20**:1297–303.

8 Danecek P, Auton A, Abecasis G, Albers CA, Banks E, DePristo MA, Handsaker RE, Lunter G, Marth GT, Sherry ST, McVean G, Durbin R, 1000 Genomes Project Analysis Group. The variant call format and VCFtools. *Bioinforma Oxf Engl* 2011;**27**:2156–8.

9 Howie BN, Donnelly P, Marchini J. A flexible and accurate genotype imputation method for the next generation of genome-wide association studies. *PLoS Genet* 2009;**5**:e1000529.

10 1000 Genomes Project Consortium, Abecasis GR, Altshuler D, Auton A, Brooks LD, Durbin RM, Gibbs RA, Hurles ME, McVean GA. A map of human genome variation from population-scale sequencing. *Nature* 2010;**467**:1061–73.

11 Genome of the Netherlands Consortium. Whole-genome sequence variation, population structure and demographic history of the Dutch population. *Nat Genet* 2014;**46**:818–25.

12 Michailidou K, Lindström S, Dennis J, Beesley J, Hui S, Kar S, Lemaçon A, Soucy P, Glubb D, Rostamianfar A, Bolla MK, Wang Q, Tyrer J, Dicks E, Lee A, Wang Z, Allen J, Keeman R, Eilber U, French JD, Qing Chen X, Fachal L, McCue K, McCart Reed AE, Ghoussaini M, Carroll JS, Jiang X, Finucane H, Adams M, Adank MA, Ahsan H, Aittomäki K, Anton-Culver H, Antonenkova NN, Arndt V, Aronson KJ, Arun B, Auer PL, Bacot F, Barrdahl M, Baynes C, Beckmann MW, Behrens S, Benitez J, Bermisheva M, Bernstein L, Blomqvist C, Bogdanova NV, Bojesen SE, Bonanni B, Børresen-Dale A-L, Brand JS, Brauch H, Brennan P, Brenner H, Brinton L, Broberg P, Brock IW, Broeks A, Brooks-Wilson A, Brucker SY, Brüning T, Burwinkel B, Butterbach K, Cai Q, Cai H, Caldés T, Canzian F, Carracedo A, Carter BD, Castelao JE, Chan TL, David Cheng T-Y, Seng Chia K, Choi J-Y, Christiansen H, Clarke CL, NBCS Collaborators, Collée M, Conroy DM, Cordina-Duverger E, Cornelissen S, Cox DG, Cox A, Cross SS, Cunningham JM, Czene K, Daly MB, Devilee P, Doheny KF, Dörk T, Dos-Santos-Silva I, Dumont M, Durcan L, Dwek M, Eccles DM, Ekici AB, Eliassen AH, Ellberg C, Elvira

M, Engel C, Eriksson M, Fasching PA, Figueroa J, Flesch-Janys D, Fletcher O, Flyger H, Fritschi L, Gaborieau V, Gabrielson M, Gago-Dominguez M, Gao Y-T, Gapstur SM, García-Sáenz JA, Gaudet MM, Georgoulias V, Giles GG, Glendon G, Goldberg MS, Goldgar DE, González-Neira A, Grenaker Alnæs GI, Grip M, Gronwald J, Grundy A, Guénel P, Haeberle L, Hahnen E, Haiman CA, Håkansson N, Hamann U, Hamel N, Hankinson S, Harrington P, Hart SN, Hartikainen JM, Hartman M, Hein A, Heyworth J, Hicks B, Hillemanns P, Ho DN, Hollestelle A, Hooning MJ, Hoover RN, Hopper JL, Hou M-F, Hsiung C-N, Huang G, Humphreys K, Ishiguro J, Ito H, Iwasaki M, Iwata H, Jakubowska A, Janni W, John EM, Johnson N, Jones K, Jones M, Jukkola-Vuorinen A, Kaaks R, Kabisch M, Kaczmarek K, Kang D, Kasuga Y, Kerin MJ, Khan S, Khusnutdinova E, Kiiski JI, Kim S-W, Knight JA, Kosma V-M, Kristensen VN, Krüger U, Kwong A, Lambrechts D, Le Marchand L, Lee E, Lee MH, Lee JW, Neng Lee C, Lejbkowicz F, Li J, Lilyquist J, Lindblom A, Lissowska J, Lo W-Y, Loibl S, Long J, Lophatananon A, Lubinski J, Luccarini C, Lux MP, Ma ESK, MacInnis RJ, Maishman T, Makalic E, Malone KE, Kostovska IM, Mannermaa A, Manoukian S, Manson JE, Margolin S, Mariapun S, Martinez ME, Matsuo K, Mavroudis D, McKay J, McLean C, Meijers-Heijboer H, Meindl A, Menéndez P, Menon U, Meyer J, Miao H, Miller N, Taib NAM, Muir K, Mulligan AM, Mulot C, Neuhausen SL, Nevanlinna H, Neven P, Nielsen SF, Noh D-Y, Nordestgaard BG, Norman A, Olopade OI, Olson JE, Olsson H, Olswold C, Orr N, Pankratz VS, Park SK, Park-Simon T-W, Lloyd R, Perez JIA, Peterlongo P, Peto J, Phillips K-A, Pinchev M, Plaseska-Karanfilska D, Prentice R, Presneau N, Prokofyeva D, Pugh E, Pylkäs K, Rack B, Radice P, Rahman N, Rennert G, Rennert HS, Rhenius V, Romero A, Romm J, Ruddy KJ, Rüdiger T, Rudolph A, Ruebner M, Rutgers EJT, Saloustros E, Sandler DP, Sangrajrang S, Sawyer EJ, Schmidt DF, Schmutzler RK, Schneeweiss A, Schoemaker MJ, Schumacher F, Schürmann P, Scott RJ, Scott C, Seal S, Seynaeve C, Shah M, Sharma P, Shen C-Y, Sheng G, Sherman ME, Shrubsole MJ, Shu X-O, Smeets A, Sohn C, Southey MC, Spinelli JJ, Stegmaier C, Stewart-Brown S, Stone J, Stram DO, Surowy H, Swerdlow A, Tamimi R, Taylor JA, Tengström M, Teo SH, Beth Terry M, Tessier DC, Thanasitthichai S, Thöne K, Tollenaar RAEM, Tomlinson I, Tong L, Torres D, Truong T, Tseng C-C, Tsugane S, Ulmer H-U, Ursin G, Untch M, Vachon C, van Asperen CJ, Van Den Berg D, van den Ouweland AMW, van der Kolk L, van der Luijt RB, Vincent D, Vollenweider J, Waisfisz Q, Wang-Gohrke S, Weinberg CR, Wendt C, Whittemore AS, Wildiers H, Willett W, Winqvist R, Wolk A, Wu AH, Xia L, Yamaji T, Yang XR, Har Yip C, Yoo K-Y, Yu J-C, Zheng W, Zheng Y, Zhu B, Ziogas A, Ziv E, ABCTB Investigators, ConFab/AOCS Investigators, Lakhani SR, Antoniou AC, Droit A, Andrulis IL, Amos CI, Couch FJ, Pharoah PDP, Chang-Claude J, Hall P, Hunter DJ, Milne RL, García-Closas M, Schmidt MK, Chanock SJ, Dunning AM, Edwards SL, Bader GD, Chenevix-Trench G, Simard J, Kraft P, Easton DF. Association analysis identifies 65 new breast cancer risk loci. *Nature* Published Online First: 23 October 2017. doi:10.1038/nature24284

13 Karczewski KJ, Francioli LC, Tiao G, Cummings BB, Alföldi J, Wang Q, Collins RL, Laricchia KM, Ganna A, Birnbaum DP, Gauthier LD, Brand H, Solomonson M, Watts NA, Rhodes D, Singer-Berk M, England EM, Seaby EG, Kosmicki JA, Walters RK, Tashman K, Farjoun Y, Banks E, Poterba T, Wang A, Seed C, Whiffin N, Chong JX, Samocha KE, Pierce-Hoffman E, Zappala Z, O'Donnell-Luria AH, Minikel EV, Weisburd B, Lek M, Ware JS, Vittal C, Armean IM, Bergelson L, Cibulskis K, Connolly KM, Covarrubias M, Donnelly S, Ferriera S, Gabriel S, Gentry J, Gupta N, Jeandet T, Kaplan D, Llanwarne C, Munshi R, Novod S, Petrillo N, Roazen D, Ruano-Rubio V, Saltzman A, Schleicher M, Soto J, Tibbetts K, Tolonen C, Wade G, Talkowski ME,

Consortium TGAD, Neale BM, Daly MJ, MacArthur DG. Variation across 141,456 human exomes and genomes reveals the spectrum of loss-of-function intolerance across human protein-coding genes. *bioRxiv* 2019;:531210.
